# Continuous successive cycles of biosorption and desorption of acid red 27 dye using water hyacinth leaves as an effective, economic, and ecofriendly biosorbent

**DOI:** 10.1007/s00449-022-02822-9

**Published:** 2022-11-27

**Authors:** Allan Eduardo Ramírez-Rodríguez, Eliseo Cristiani-Urbina, Liliana Morales-Barrera, Erick Aranda-García

**Affiliations:** Departamento de Ingeniería Bioquímica, Instituto Politécnico Nacional, Escuela Nacional de Ciencias Biológicas, Avenida Wilfrido Massieu S/N, Unidad Profesional Adolfo López Mateos, Delegación Gustavo A. Madero, Ciudad de México, 07738 México

**Keywords:** Acid red 27, Biosorption, Desorption, Packed-bed column, Sodium bicarbonate, Water hyacinth leaf

## Abstract

We investigated the capacity of water hyacinth leaves (LEC) to biosorb 75 mg/L acid red 27 (AR27) in a continuous system comprising 30 successive biosorption/desorption cycles in a packed-bed column at pH 2.0 and 56.5 L/m^2^·h volumetric flux. Using 0.025 M NaHCO_3_ eluent at 113 L/m^2^·h volumetric flux, all the dye was desorbed (100% desorption efficiency) from the loaded LEC biomass within 5–6 h. The same biosorbent was used for 147.5 consecutive days. The AR27 biosorption capacity, breakthrough time, and exhaustion time decreased from 69.4 to 34.5 mg/g, 74.81 to 14.1 h, and 101.1 to 34.1 h, respectively, and the critical bed height increased from 1.04 to 2.35 cm, as the number of biosorption/desorption cycles increased from 1 to 30. LEC life factor based on biosorption capacity predicted that the packed bed would be exhausted after 51.95 cycles. LEC is a promising biosorbent for bioremediation of AR27-laden wastewaters.

## Introduction

Global population growth, socioeconomic, scientific, and technological development, and industrialization have resulted in the discharge of toxic azo dye-laden industrial effluents into receiving waters. Consequently, aquatic flora and fauna have been negatively impacted, water has become unfit for human consumption, and public health is threatened [1–3]. The removal of azo dyes from industrial wastewaters is a critical environmental issue that has attracted substantial research attention in recent years [4].

Biosorption is a cheap, simple, effective, efficient, safe, and sustainable environmental remediation technology. It has proven to be superior to conventional physicochemical and other biological methods for removing toxic azo dyes from aquatic ecosystems [5, 6].

Biosorption has demonstrated great potential for dye recovery [7]. Biosorbents can be regenerated, recycled, and reused in multiple successive dye biosorption/desorption cycles. These properties lower treatment costs, mitigate the environmental impact, reduce the amount of biosorbent required, and decrease waste generation. Furthermore, as the recovered dyes are reusable, they can be sold as valuable commodities, thereby offsetting the costs and enhancing the profitability of dye-contaminated wastewater treatment [8–10].

However, most previous studies evaluated the environmental factors that affect biosorbent capacity in batch dye biosorption. In contrast, little is known about biosorbent desorption, regeneration, and recycling in the successive dye biosorption/desorption cycles of continuous systems. However, these characteristics may, in fact, determine the feasibility and practicality of industrial-scale biosorption [11, 12]. Therefore, the mechanisms of dye desorption and biosorbent regeneration must be elucidated to make continuous dye bioremediation a viable process.

Amaranth azo dye (acid red 27; AR27) commonly occurs in wastewater generated by the food, textile, leather, paper, beverage, confectionery, photography, cosmetic, pharmaceutical, and phenol–formaldehyde resin industries [13–16]. At concentrations > 0.15 mg/kg, AR27 may cause acute and chronic toxicity, allergies, respiratory problems, birth defects, and tumors in humans [15, 17]. Moreover, AR27 is cytostatic/cytotoxic, an endocrine disruptor, mutagenic, teratogenic, genotoxic, embryotoxic, and carcinogenic [14, 17–20]. For these reasons, AR27 has been banned by the United States Food and Drug Administration (USFDA) and its use is restricted in many other countries worldwide [14, 15, 21, 22].

Previous studies showed that the leaves of water hyacinth (*Pontederia crassipes*) (LEC) effectively biosorb AR27 dye from aqueous solutions in both batch and continuous systems [23–25]. In addition, this dye can be recovered with 100% desorption efficiency from the loaded LEC biomass by using 0.025 M NaHCO_3_ as the eluent/desorbent. The desorbed LEC can then be reused in at least seven batch AR27 biosorption/desorption cycles without losing its bioremediation efficacy or undergoing any structural damage [20].

However, in spite of its great importance and relevance for the processes of adsorbate recovery and biosorbent regeneration in large-scale applications, there is no information in the literature regarding the effect of volumetric flux of the eluent on the desorption of an adsorbate or on the regeneration, recycling, and reuse of a biosorbent/adsorbent in multiple successive AR27 biosorption/desorption cycles in continuous systems. This study attempts to fill these research gaps.

The main objectives of this work are: (1) to assess the effect of eluent solution (0.025 M NaHCO_3_) volumetric flux on AR27 desorption, and (2) to evaluate the performance of LEC at removing AR27 dye from aqueous solutions in 30 successive biosorption/desorption cycles in an up-flow packed-bed column reactor, by using the optimal eluent volumetric flux.

## Materials and methods

### Biosorbent

*Pontederia crassipes* (water hyacinth; formerly *Eichhornia crassipes*) plants were harvested from the Xochimilco Canals (19°15′30.7″N, 99°05′00.3″W) in Mexico City, Mexico. The leaves were excised, washed with tap water to remove water-soluble impurities and adhering particles, and rinsed with deionized water. They were then cut into small pieces, dried in a oven at 60 °C for 36 h, and ground in a mill. The leaf powder was then passed through stainless steel ASTM sieves to obtain particles 0.15–0.3 mm in size. They were designated LEC and stored in airtight glass bottles until their use in the following AR27 biosorption and desorption experiments.

### Chemical reagents and analytical methods

To prepare 3 g/L AR27 (purity ≥ 95%; Sigma-Aldrich Corp., St. Louis, MO, USA) stock solution, a certain amount of dye was accurately weighed and dissolved in deionized water. A 75 mg/L AR27 test solution was prepared by diluting the AR27 stock solution with deionized water. Its pH was adjusted with 0.1 M HCl to 2.0 which is the optimum for AR27 biosorption by LEC in a continuous system with a packed-bed column reactor [25]. The AR27 concentration was determined at 520 nm in a Thermo Scientific UV–Vis Evolution 201 spectrophotometer (Thermo Fisher Scientific, Waltham, MA, USA) [23].

### Packed-bed column reactor

**Fig. 1:**
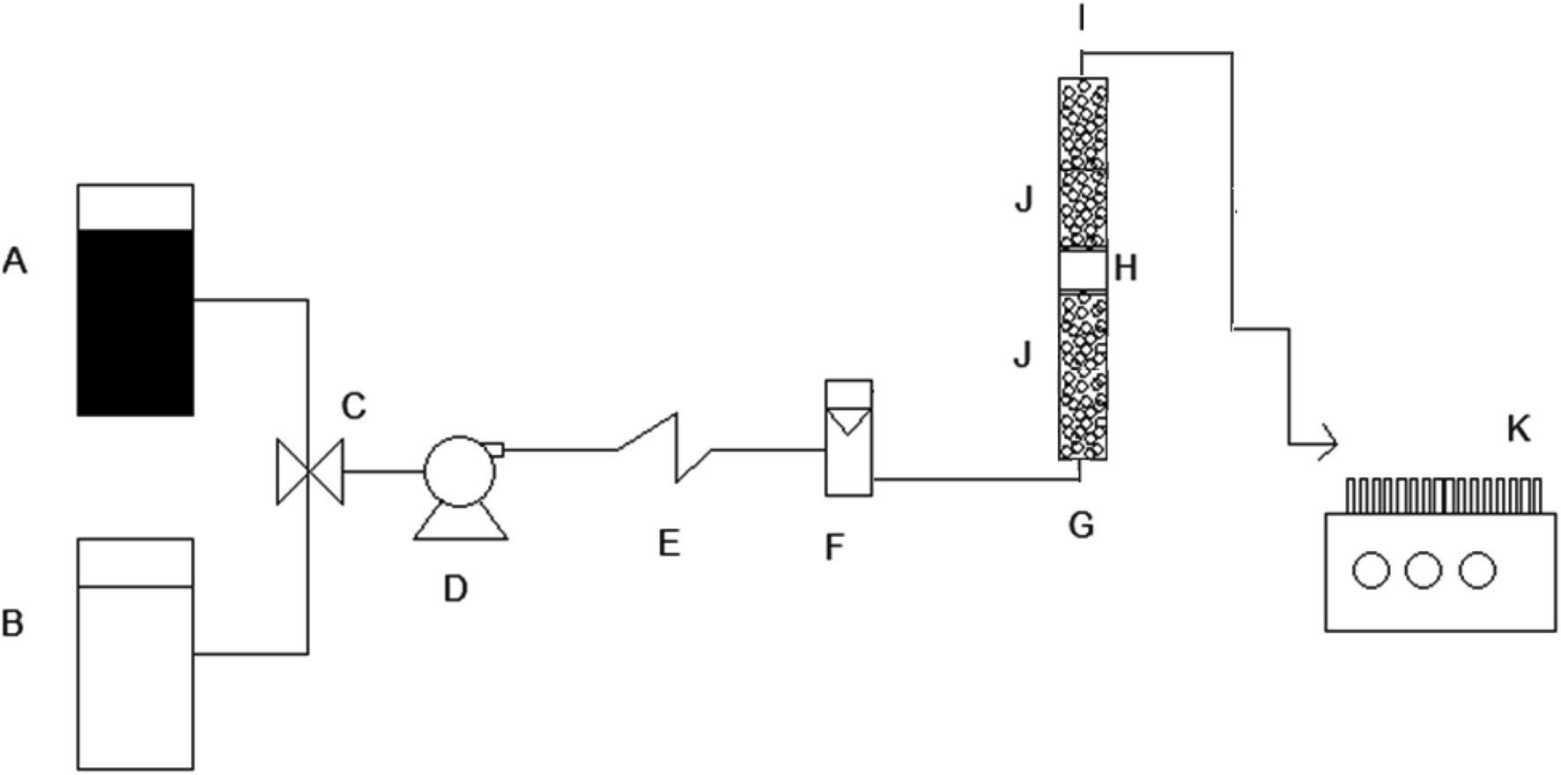
Schematic diagram of packed-bed column system (A, AR27 solution reservoir; B, eluent solution (0.025 M NaHCO_3_) reservoir; C, three-way valve; D, peristaltic pump; E, pulse dampener; F, flowmeter; G, cylindrical column; H, LEC packed bed; I, effluent; J, glass beads; K, fraction collector

Figure 1 is a schematic representation of the experimental system setup used in the present study.

Continuous-flow AR27 biosorption and desorption experiments were conducted in a cylindrical glass column (i.d. = 1.3 cm, *h* = 20 cm) at an average temperature of 20 ± 2 °C. The column diameter:particle diameter ratio was set to >10 to avoid wall effects that may alter the shape and slope of the breakthrough curve [26].

The column was packed with a known mass of LEC between two layers of glass beads (*d* = 0.3 cm) and two 200-mesh stainless steel sieves. The glass beads under the LEC bed supported it and ensured even distribution of the AR27 inlet solution over the entire cross-section of the cylindrical column. The glass beads over the LEC bed prevented LEC loss. The 200-mesh stainless steel sieves prevented biosorbent loss. The LEC packed-bed porosity (*ε*_*b*_) and density (*ρ*_b_) were ~25% and 0.188 g/cm^3^, respectively [25].

NaHCO_3_ eluent (0.025 M, pH 8.0, and volumetric flux of 37.67, 56.5, 75.34 or 113 L/m^2^·h) or influent AR27 solution (75 mg/L, pH 2.0, and volumetric flux = 56.5 L/m^2^·h) were introduced at the base of the column through a sintered glass diffuser, which enhanced fluid dispersion and distribution within the column. The eluent or AR27 influent passed through the LEC packed bed.

Effluent exiting the top of the column was collected with a programmable LKB Bromma fraction collector (LKB Bromma Ltd., Sollentuna, Sweden). The effluent was centrifuged at 5000 rpm for 4 min and the AR27 concentration in the supernatant was determined spectrophotometrically at 520 nm.

### Effect of volumetric flux of eluent on AR27 desorption in packed-bed column

A previous study on a batch system established that 0.025 M NaHCO_3_ was the optimal eluent concentration for the desorption of AR27-loaded LEC biomass [20]. In the present work, then, continuous AR27 desorption was conducted in a packed-bed column and the effects of the volumetric flux of 0.025 M NaHCO_3_ at 37.67, 56.5, 75.34, and 113 L/m^2^·h on dye desorption were evaluated. These volumetric fluxes correspond to volumetric flow rates of 5, 7.5, 10, and 15 mL/h, respectively.

The LEC packed bed in the column was loaded with AR27 before conducting the desorption assays. An influent AR27 solution with pH, concentration, and volumetric flux of 2.0, 75 mg/L, and 56.5 L/m^2^·h, respectively, was continuously fed into a column packed with 1.5 g LEC until the influent and effluent AR27 concentrations were equal. The AR27-loaded LEC packed bed was washed with deionized water to remove any free AR27. Then, 0.025 M NaHCO_3_ was passed through the packed-bed column at one of the aforementioned volumetric fluxes to desorb the dye from the AR27-loaded LEC packed bed. The eluent volumetric flux optimally desorbing the AR27 from the LEC packed bed was selected for the subsequent studies.

### Multiple consecutive AR27 biosorption/desorption cycles in a packed-bed column reactor

To assess LEC regeneration and recycling performance, 30 sequential AR27 biosorption/desorption cycles were evaluated for the same biosorbent in a packed-bed column reactor.

The AR27 biosorption assays were conducted by feeding 75 mg/L AR27 solution (pH 2.0) through a 4-cm packed bed containing 1 g LEC. The up-flow mode was used and the volumetric flux was 56.5 L/m^2^·h. According to Ramírez-Rodríguez et al. [25], these operating conditions were deemed optimal for continuous AR27 biosorption in the LEC packed-bed column as they realized the highest experimental AR27 biosorption capacity and the longest column service time. Effluent samples were collected at regular intervals and their residual AR27 concentrations were measured. AR27 flux through the column was stopped when the effluent and influent AR27 concentrations were equal. This condition indicated no further AR27 biosorption.

The AR27-loaded LEC biomass was then regenerated with 0.025 M NaHCO_3_ at the previously selected volumetric flux rate. After AR27 desorption, the LEC bed was washed with deionized water until the effluent had the same pH as the rinse. The regenerated LEC bed was then used in the subsequent AR27 biosorption cycle to determine whether the same LEC could retain its efficacy over multiple sequential cycles. Thirty successive AR27 biosorption/desorption cycles were tested here.

### Evaluation of LEC packed-bed column performance at AR27 biosorption/desorption

The performance of the LEC packed-bed column at AR27 biosorption was evaluated using breakthrough curves established by plotting the ratio between the outlet AR27 concentration (*C*_t_, mg/L) and the inlet AR27 concentration (*C*_o_, mg/L) as a function of column operating time (*t*, h); (*C*_t_/*C*_o_ vs. *t*).

The amount of AR27 biosorbed by the LEC packed bed (*m*_T_, mg) was calculated as follows [27]:

1 $$m_{\rm T} = \frac{{C_{0} F}}{1000}\int_{t = 0}^{{t = t_{\rm T} }} {\left( {1 - \frac{{C_{\rm t} }}{{C_{0} }}} \right)dt} ,$$

where *F* is the volumetric flow rate (mL/h) of the inlet AR27 solution and *t*_T_ is the total operating time (h) of the packed-bed column in the biosorption cycle.

The (specific) biosorption capacity (*q*_*b*_) (mg/g) of AR27 was estimated as follows [27]:

2 $${q}_{\mathrm{b}}=\frac{{m}_{\mathrm{T}}}{{m}_{\mathrm{b}}},$$

where *m*_b_ is the LEC biomass (g) packed in the column.

The breakthrough time (*t*_br_) is the point at which the effluent adsorbate concentration reaches a predetermined or recommended level [26]. In the present study, 1 mg/L effluent AR27 was selected for *t*_br_ as this concentration is highly visible in water [28]. The bed exhaustion time (*t*_ex_) is the point at which the effluent and influent adsorbate concentrations are equal (*C*_t_/*C*_o_ = 1.0). At *t*_ex_, the bed is saturated with adsorbate and is no longer effective [26]. Here, *t*_ex_ was the point at which the effluent AR27 concentration was 95% of the influent concentration (*C*_*t*_/*C*_o_ = 0.95). Hence, *C*_t_ = 71.25 mg/L.

The metric length of the biosorption zone (*H*_min_) is also known as the critical or minimum bed length. It represents the minimum biosorbent bed length required to attain *t*_br_ at *t* = 0 [29] and was estimated as follows [30]:

3 $$H_{\min } = H\left( {1 - \frac{{t_{br} }}{{t_{ex} }}} \right),$$

where *H* is the height of the LEC bed, namely, 4 cm.

The mass of AR27 desorbed from the AR27-loaded LEC packed bed (*m*_D_, mg) was calculated as follows:

4 $$m_{\rm D} = \frac{{F_{\rm D} }}{1000}\int_{{t_{\rm D} = 0}}^{{t_{\rm D} = t_{\rm TD} }} {C_{\rm tD} dt_{\rm D} } ,$$

where *F*_D_ is the volumetric flow rate of the desorbing solution (0.025 M NaHCO_3_), *C*_tD_ is the concentration (mg/L) of desorbed AR27, and *t*_TD_ (h) is the total desorption time.

The desorbing efficiency (*E*_D_, %) was calculated as follows:

5 $$E_{\rm D} = 100\left( {\frac{{m_{\rm D} }}{{m_{\rm T} }}} \right).$$

### Statistical analysis

Three independent continuous AR27 biosorption/desorption assays were conducted to validate accuracy and reproducibility within 4% error. The data are presented as average values. Data for continuous AR27 biosorption/desorption were statistically analyzed by two-way ANOVA with Tukey’s test at 95% confidence level in GraphPad Prism v. 8.4 (GraphPad Software, La Jolla, CA, USA).

## Results and discussion

### Effect of eluent volumetric flux on AR27 desorption

**Fig. 2:**
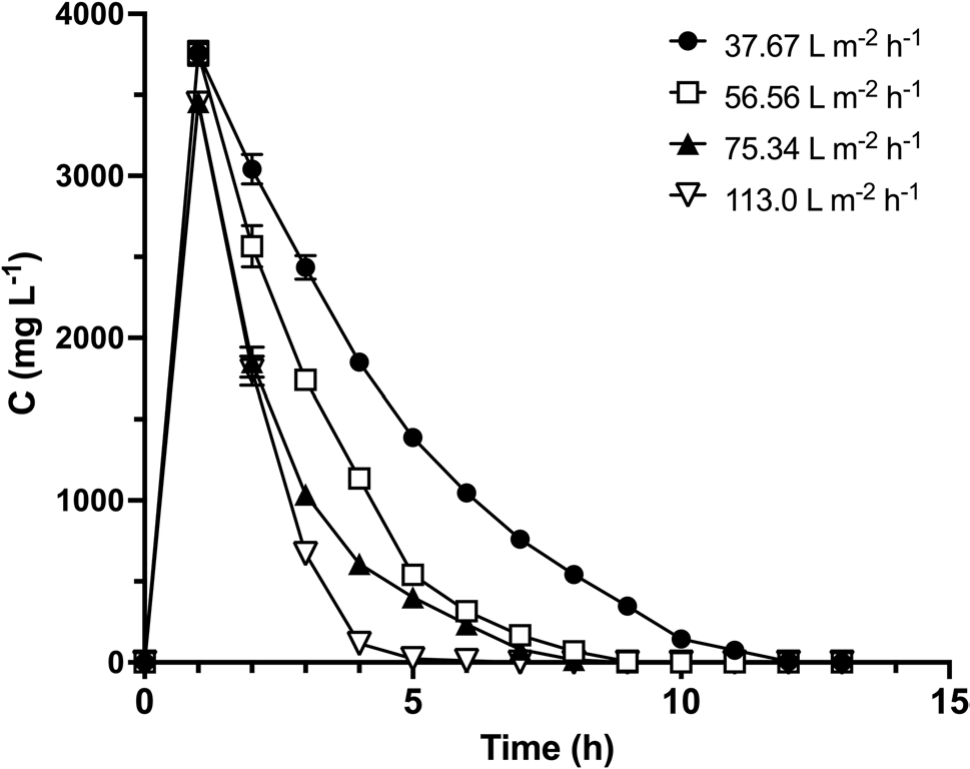
Elution curves for AR27 desorption from loaded LEC at various volumetric fluxes of eluent

An effective biosorbent for the removal of dyes from aqueous solutions must exhibit both good biosorption and dye desorption performance [11]. Hence, we investigated the effect of the volumetric flux of the eluent solution (0.025 M NaHCO_3_) on AR27 desorption from loaded LEC and selected the most suitable volumetric flux. Figure 2 shows the AR27 elution curves obtained at various volumetric fluxes of the eluent.

The elution curves showed that AR27 desorption had a similar trend at all volumetric fluxes. The eluted AR27 concentration displayed a rapid initial increase until it reached a maximum (*C*_p_). It then gradually decreased with increasing desorption time until the AR27 concentration reached zero. The time required to completely desorb the AR27 dye decreased from 13 to 6 h as the volumetric flux of the eluent solution increased from 37.67 to 113 L/m^2^·h.

**Fig. 3:**
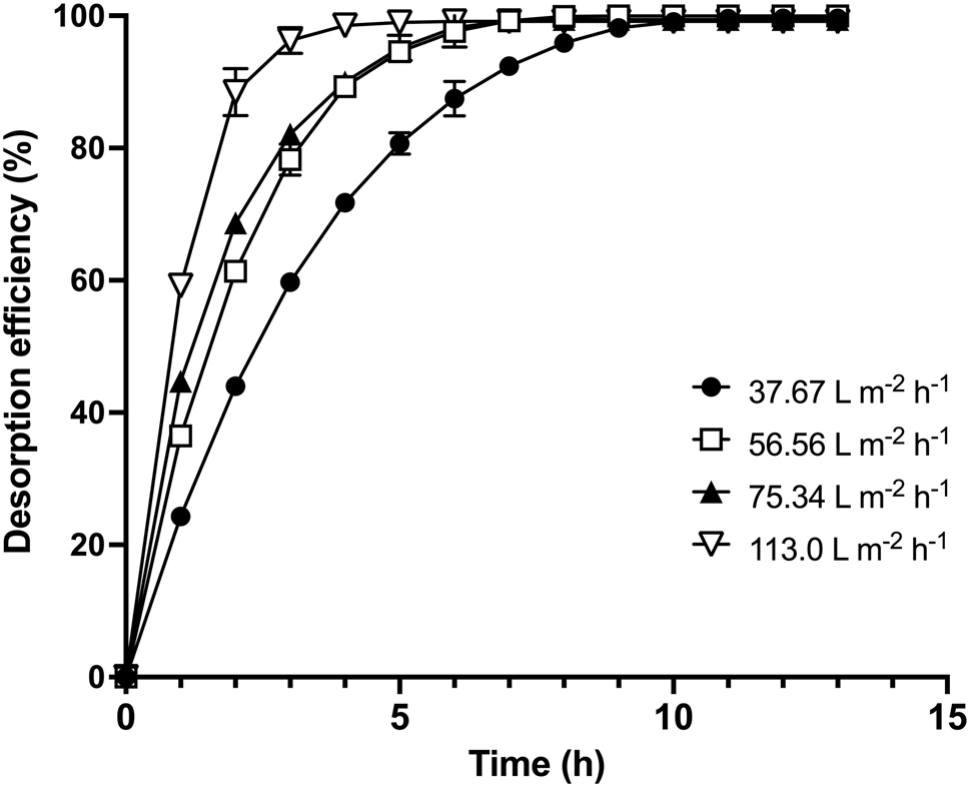
AR27 desorption efficiency at various volumetric fluxes

Figure 3 shows the variation in AR27 desorption efficiency with desorption time. The desorption efficiency depended on the volumetric flux of the eluent. At all volumetric fluxes tested, the desorption efficiency gradually increased with desorption time until it reached 100%. The time required to reach 100% desorption efficiency decreased from 13 to 6 h as the volumetric flux increased from 37.67 to 113 L/m^2^·h. This was possibly due to the fact that the rate of mass transfer of the AR27 molecules from the LEC packed bed to the fluid, increased with volumetric flux, because of a greater thermodynamic driving force for dye desorption. The desorption times were lower than those reported previously for the biosorption of AR27 by LEC packed-bed under different operating conditions [25].

Prolonged contact between the eluent and the biosorbent packed bed during regeneration may damage the biosorption-binding sites [26, 31], and can consequently decrease the biosorption capacity of the packed bed in subsequent biosorption cycles. We thus selected a volumetric flux of 113 L/m^2^·h for the subsequent assays, as this would be able to desorb all dye from the AR27-loaded LEC packed bed in the shortest time.

To the best of our knowledge, this work is the first to report the effect of eluent volumetric flux on the desorption of an adsorbate.

### Multiple consecutive AR27 biosorption/desorption cycles in a packed-bed column

Successive biosorbent regeneration and reuse is essential in industrial dye biosorption from polluted effluents. This approach lowers treatment costs, reduces continuous biosorbent dependence, and improves dye recovery [31]. We conducted LEC regeneration and reuse assays to compare the continuous AR27 biosorption performance of a packed-bed column undergoing 30 biosorption/desorption cycles.

### Biosorption curves

**Fig. 4:**
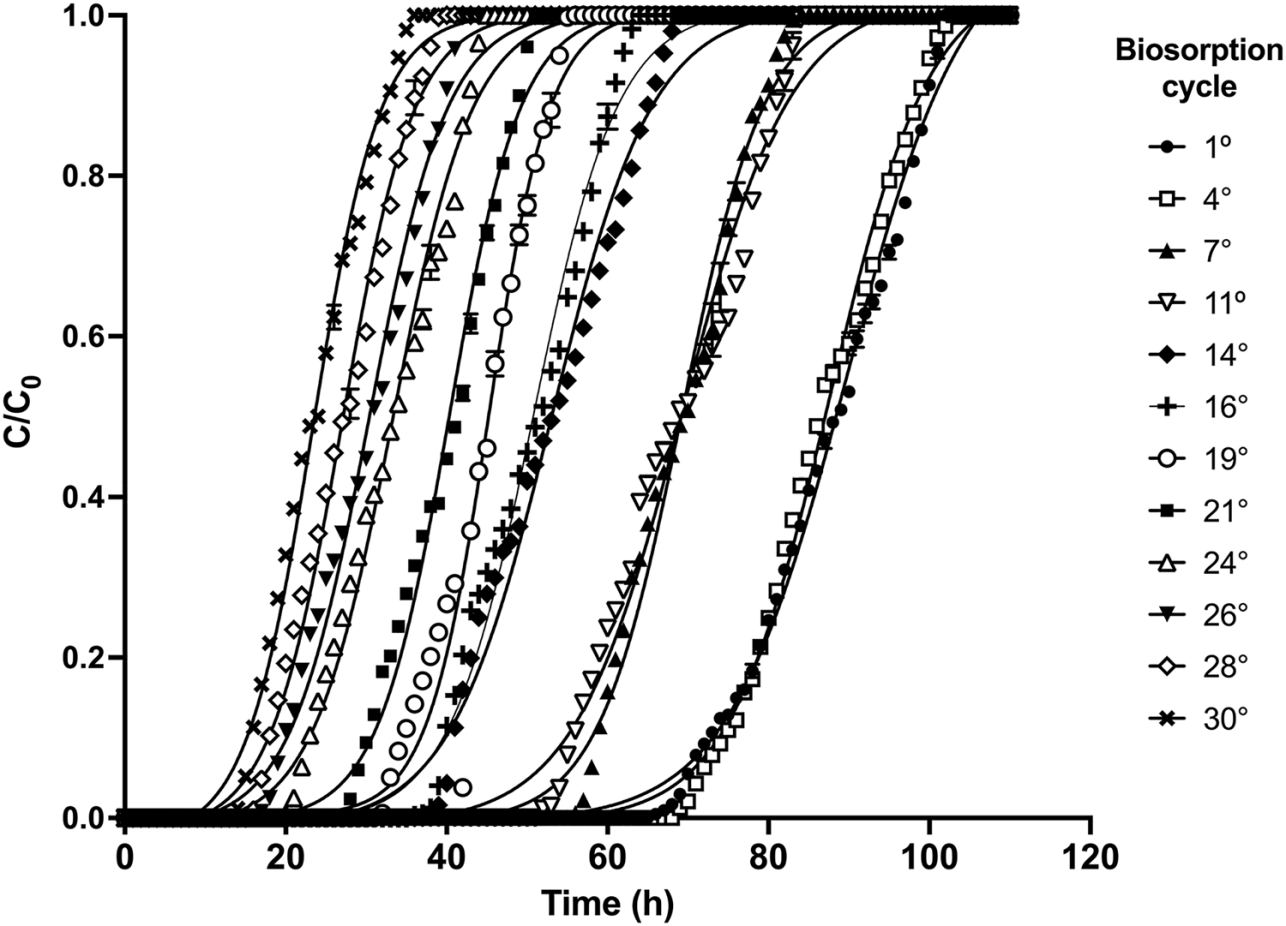
Breakthrough curves for AR27 biosorption onto LEC over 30 biosorption/desorption cycles

Figure 4 shows some breakthrough curves obtained for the AR27 biosorption cycles. Evidently, no measurable concentration of AR27 was found in the outlet effluent for any cycle in the first hours of operation of the packed-bed column; thereafter, the AR27 effluent concentrations increased progressively as the operation time increased, indicating a possible decrease in the removal capacity of AR27 due to the gradual exhaustion of binding sites for biosorption of AR27.

The breakthrough curves of cycles 1–6 and those of cycles 7–11 were similar in shape and slope. However, the latter had slightly greater slopes than the former. The slopes of the breakthrough curves increased as the number of cycles increased from 12 to 30. Furthermore, as the number of biosorption cycles increased, the breakthrough curves shifted from right to left. These results indicate that the effluent AR27 concentrations and the rate of packed-bed exhaustion increased while the number of AR27 dye molecules removed by the LEC packed bed decreased with increasing number of biosorption cycles.

**Fig. 5:**
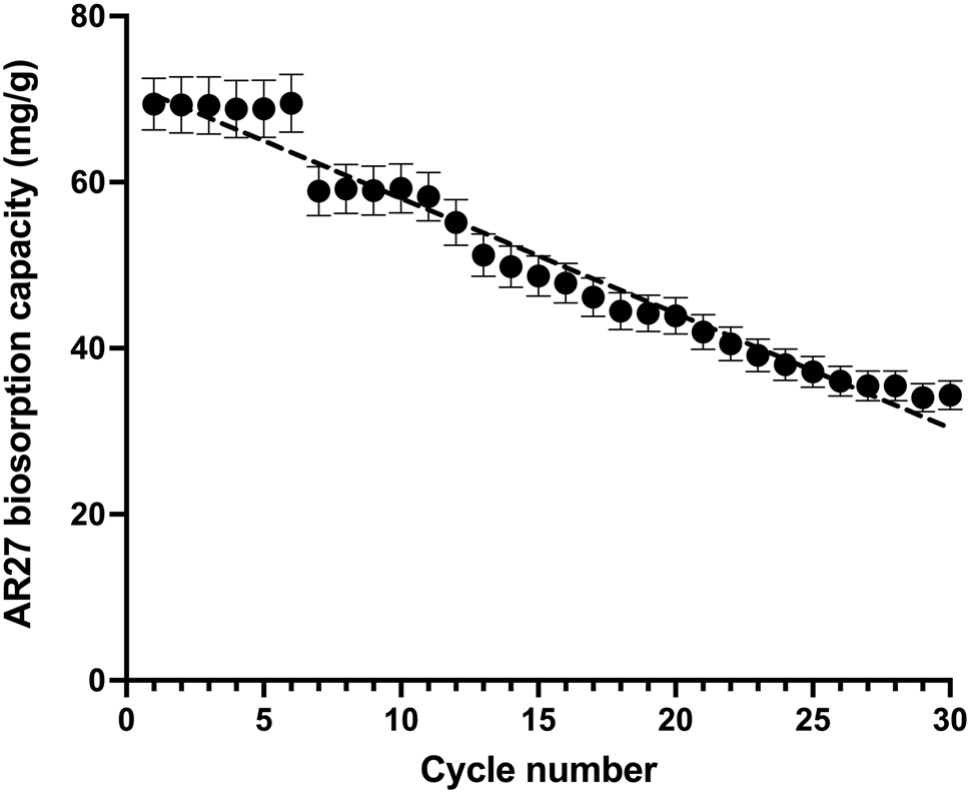
AR27 biosorption capacity as a function of biosorption/desorption cycle number

The biosorption capacities of AR27 over the 30 biosorption/desorption cycles are shown in Fig. 5. They were deemed "effective" as they were related to the initial LEC mass.

The AR27 biosorption capacity was virtually constant over the first six biosorption cycles (*p* > 0.05) and its average was 69.18 mg/g. The biosorption capacity then decreased, remained nearly constant (~58.92 mg/g) over cycles 7–11 (*p* > 0.05), and gradually decreased from 58.92 to 34.5 mg/g over cycles 11–30 (Fig. 5). The AR27 biosorption capacities of cycles 7–11, 12, 16, 22, and 30 were ~15, 20, 30, 40, and 50% lower, respectively, than those of cycles 1–6.

In general, a negative linear dependence of AR27 biosorption capacity on the number of biosorption/desorption cycles was apparent (straight line in Fig. 5), indicating that AR27 biosorption capacity decreased proportionally as the number of biosorption/desorption cycles increased.

Reck et al. [32] reported a 68.25% decrease in biosorption capacity over four tartrazine yellow biosorption/desorption cycles in a packed-bed column containing *Moringa oleifera* seed biosorbent. The capacity of dead *Candida tropicalis* cells immobilized on calcium alginate beads to remove synthetic textile dyes decreased by ~84% over eight biosorption/desorption cycles [31]. Sana and Jalila [11] studied methylene blue removal in columns packed with sheep manure waste, sawdust, or internal almond shells and observed decreases in dye biosorption capacity of ~56.25, 69.6, or 5.46%, respectively, over two biosorption/desorption cycles.

Decreases in biosorption capacity within increasing number of biosorption/desorption cycles may be caused by changes in biosorbent structure and chemistry as well as mass transfer and flow conditions within a packed-bed column [33]. Biosorption capacity may also deteriorate because of a reduction in the number of active biosorbent dye-binding sites [32]. Additionally, trace contaminants in the influent AR27 solution and/or NaHCO_3_ eluent may accumulate on the LEC biomass and block or destabilize the active biosorption sites [33], causing a decrease in biosorption capacity.

Here, it was observed that the height of the LEC packed-bed was not constant over the 30 biosorption/desorption cycles. Its initial height was 4 cm but it decreased to 3.8, 3.6, 3.4, 3.3, and 3.2 cm at the end of cycles 7, 13, 19, 25, and 30, respectively. Hence, there was a 20% decrease in packed bed height over the 30 column operation cycles, which may partially explain the observed decrease in AR27 biosorption capacity. Loss of packed bed height and biosorbent biomass over multiple biosorption/desorption cycles was previously reported [33].

**Fig. 6:**
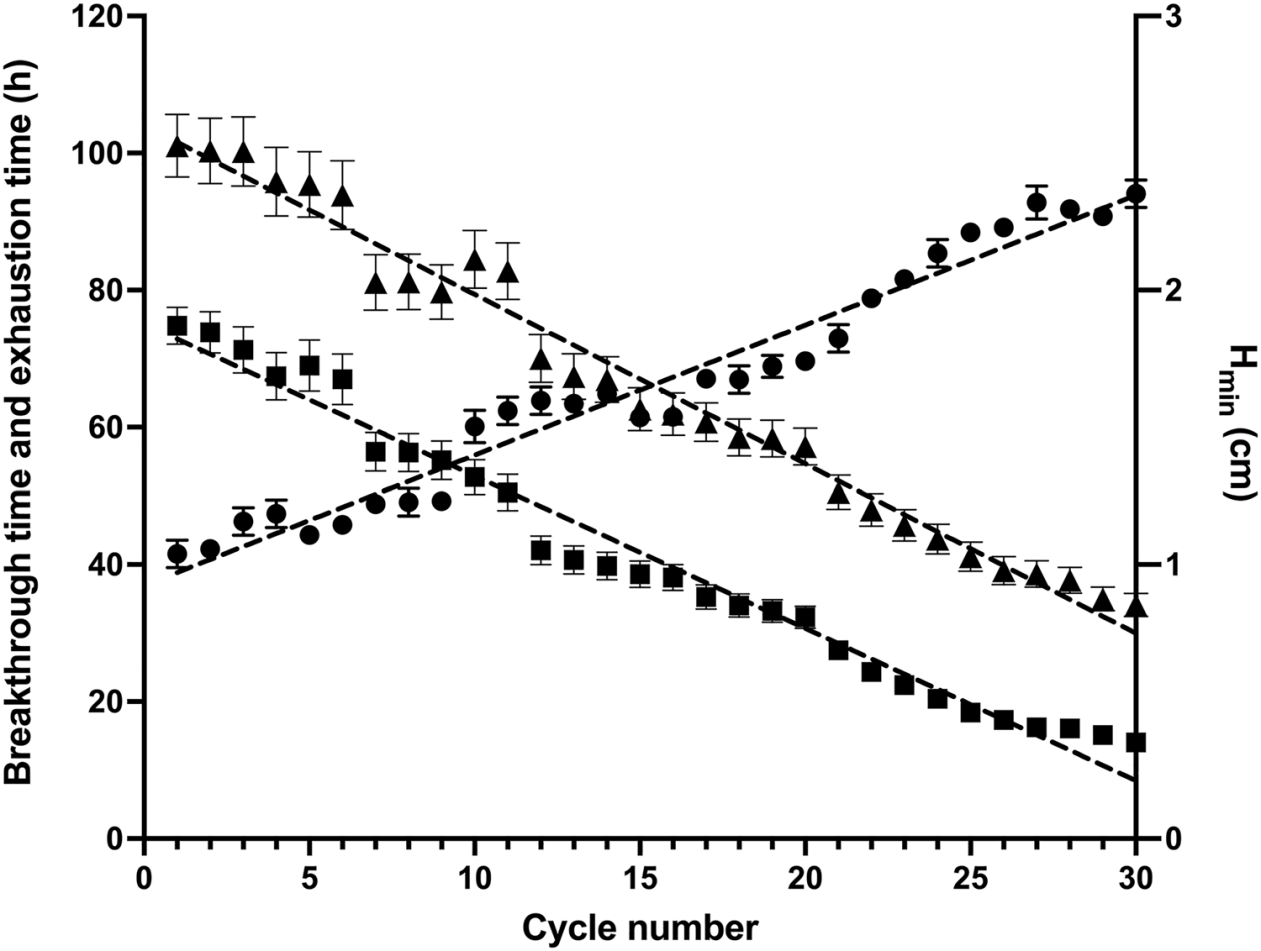
Variations in breakthrough time, exhaustion time, and critical bed height with cycle number (filled square, breakthrough time; filled triangle, exhaustion time; filled circle, critical bed height)

The breakthrough (*t*_br_) and exhaustion (*t*_ex_) times of the LEC packed-bed column decreased linearly as the number of biosorption/desorption cycles increased (Fig. 6). These results indicate that breakthrough and exhaustion times became shorter as the number of biosorption/desorption cycles increased, which may be due to the progressive reduction in the number of active biosorption sites of AR27, which in turn, caused the decrease in the AR27 biosorption capacity.

The aforementioned behavior was reported in several earlier biosorption/desorption cycle studies [31–33]. It was attributed to gradual deterioration of the active biosorption sites after their repeated use over successive biosorption/desorption cycles [26].

The critical or minimum bed height (*H*_min_) evaluates the mass transfer zone in terms of packed bed height and is useful for adsorption scale-up in packed-bed column systems [33]. In the present work, *H*_min_ increased linearly with the number of biosorption/desorption cycles (Fig. 6). These results suggest that the mass transfer zone increased, and that the service time of the LEC packed-bed column and the portion of fully utilized LEC biosorbent inside the column decreased as the number of biosorption/desorption cycles increased. Volesky et al. [33] reported qualitatively similar behavior for copper biosorption in a column packed with *Sargassum filipendula*.

The rate at which the performance of the LEC packed bed for AR27 biosorption declines may be established in terms of activity indicators or "life factors" based on the bed biosorption capacity, breakthrough time, or critical bed height with respect to the number of biosorption/desorption cycles [29, 33].

There were linear relationships among bed biosorption capacity (Fig. 5), breakthrough time, critical bed height (Fig. 6), and number of biosorption/desorption cycles:

6 $$q_{\rm b} = q_{b,0} + k_{\rm q} n$$

7 $${t}_{\mathrm{br}}= {t}_{\mathrm{br},\mathrm{o}}+{k}_{b}n$$

8 $${H}_{\mathrm{min}}= {H}_{\mathrm{min},\mathrm{o}}+{k}_{H}n,$$

where *q*_b,o_ (mg/g), *t*_b*r,o*_ (h), and *H*_min,o_ (cm) are the initial bed biosorption capacity, breakthrough time, and critical bed height, respectively, *k*_q_ (mg/g/cycle), *k*_b_ (h/cycle), and *k*_H_ (cm/cycle) are the corresponding life factors, and *n* is the number of biosorption/desorption cycles.

The following expressions were formulated to determine AR27 biosorption by the LEC packed bed:

9 $${q}_{\rm b}=71.894-1.384n \qquad {R}^{2}=0.9675$$

10 $${t}_{\mathrm{br}}=75.111-2.2213n \qquad {R}^{2}=0.9764$$

11 $${H}_{\mathrm{min}}=0.9238+0.0474n \qquad {R}^{2}=0.9611,$$

where *R*^2^ is the determination coefficient. Equations 9, 10, and 11 established that the LEC packed bed would be completely exhausted (*q*_b_ = 0 mg/g) after 51.95 biosorption/desorption cycles, that *t*_br_ = 0 h would be reached at cycle 33.81, and that *H*_min_ would be 3.386 cm at cycle 51.95, respectively.

As shown above, the AR27 biosorption capacity of LEC decreased with increasing number of biosorption/desorption cycles. Nevertheless, even the lowest biosorption capacity of LEC in a continuous system (34.35 mg/g, cycle 30) was higher than those of alumina-reinforced polystyrene [34], de-oiled soya, bottom-ash [35], and peanut hull [36] in batch systems. Therefore, LEC is a highly effective as a biosorbent removing AR27 from aqueous solutions. Moreover, it is an abundant, readily available, inexpensive, and environmentally friendly biomaterial.

### Desorption curves

In successive biosorption/desorption cycles, the performance of the biosorption steps is contingent upon the efficiency of the preceding desorption steps [29, 33]. Biosorbent regeneration is essential for cost-effective wastewater treatment by biosorption technology.

**Fig. 7:**
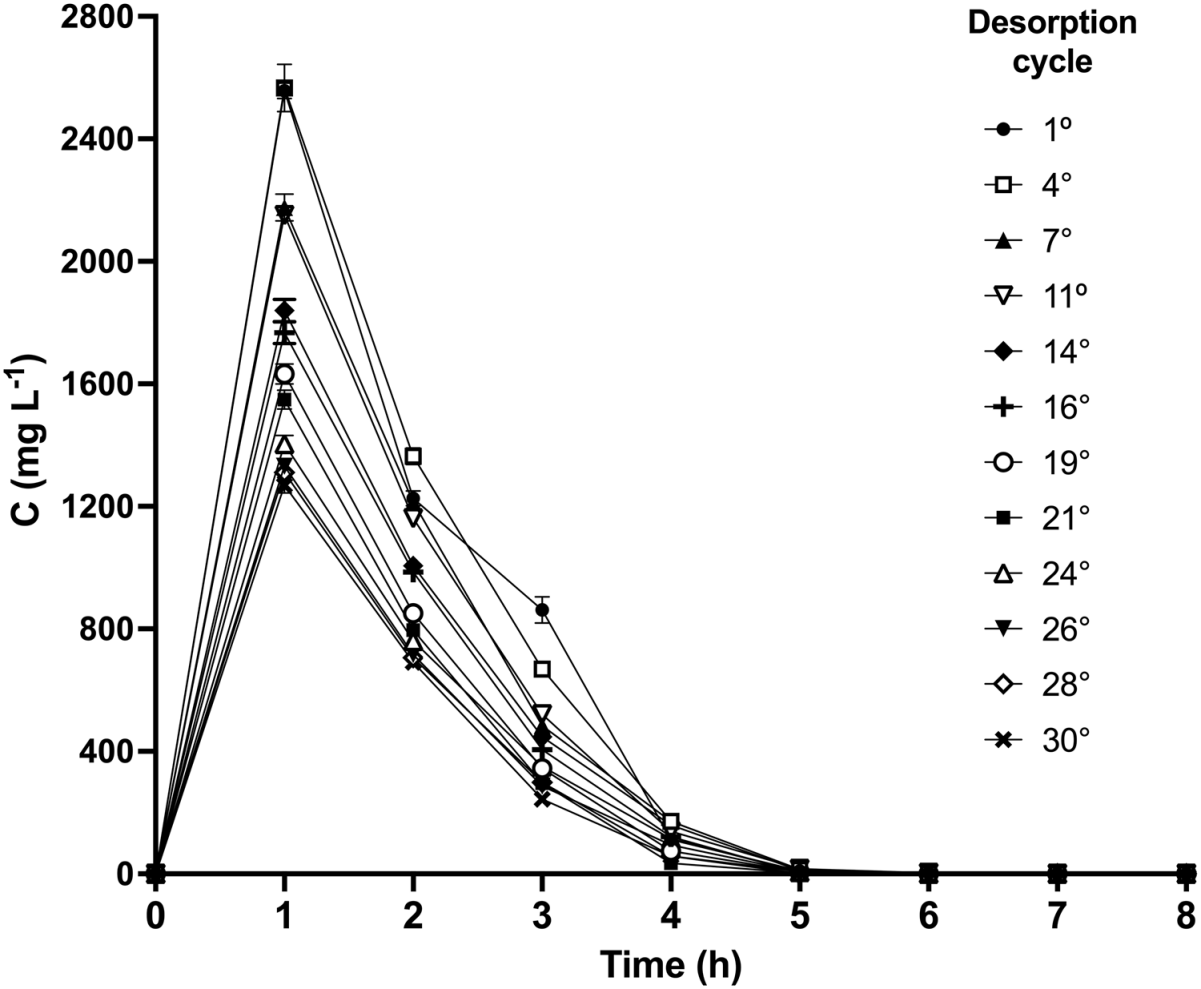
Elution curves for desorption from AR27-loaded LEC over 30 biosorption/desorption cycles

To regenerate the LEC biosorbent, an AR27 desorption step was performed after each biosorption step when the LEC packed-bed column was saturated with dye. Figure 7 shows the AR27 elution curves over several biosorption/desorption cycles in the LEC packed-bed reactor.

Similar AR27 elution profiles were observed for all cycles. The AR27 elution curves showed that the concentration of desorbed AR27 rapidly increased at first, reached a maximum (peak) value (*C*_p_), and gradually decreased to zero as desorption progressed. Total AR27 elution was completed within 5 to 6 h, meaning that the desorption process was faster than the biosorption process.

**Fig. 8:**
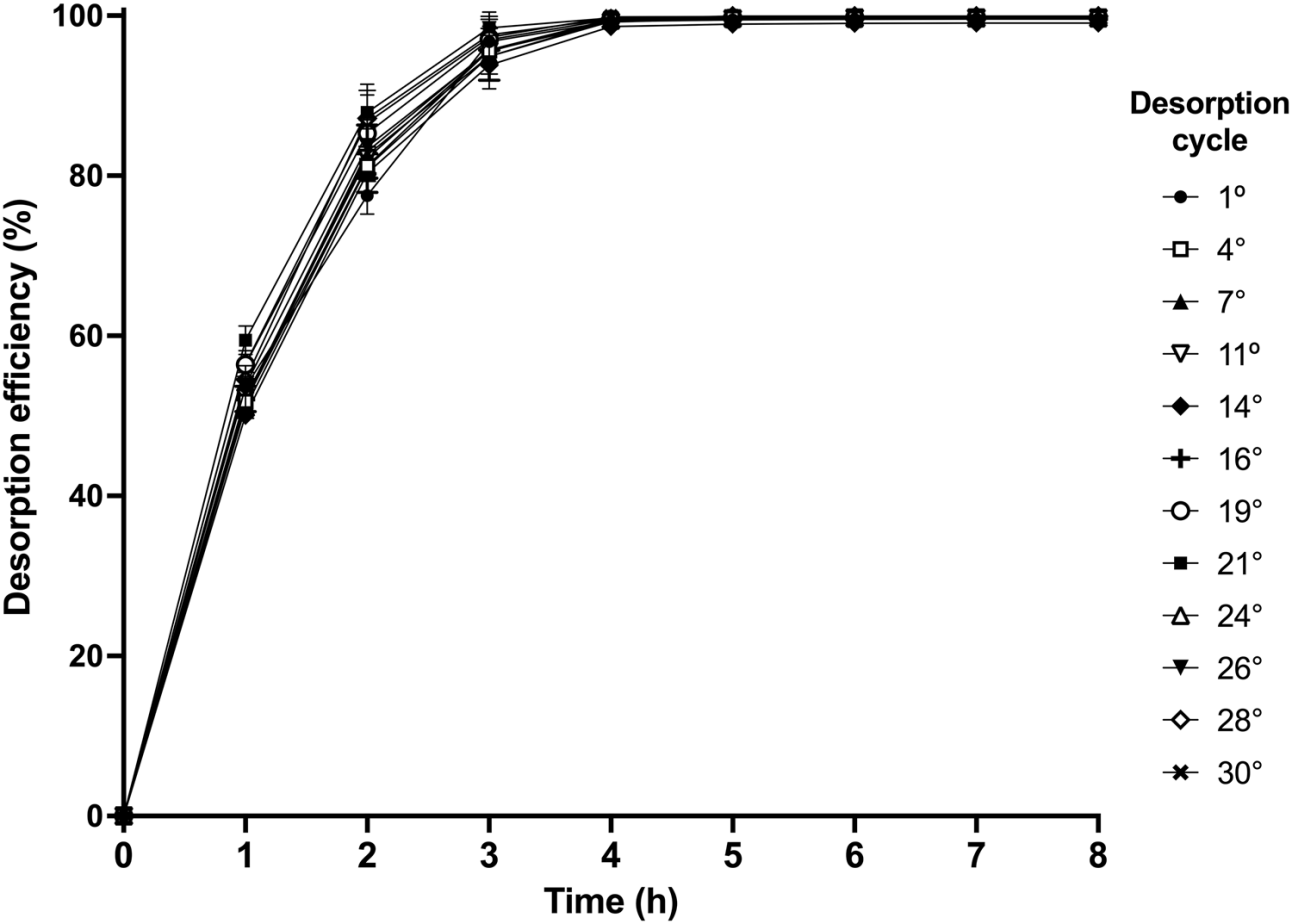
AR27 desorption efficiency with respect to desorption time over successive biosorption/desorption cycles

*C*_p_ remained constant over the first cycles and decreased thereafter. In contrast, the time (*t*_p_) to maximum desorbed dye concentration (*C*_p_) was 1 h and it was the same for all cycles. The AR27 desorption efficiency gradually increased with desorption time until it reached 100% in all cycles (Fig. 8).

AR27 elution with 0.025 M NaHCO_3_ was rapid and resulted in a concentrated AR27 solution within a small eluent volume, which is convenient for the easy and cost-effective recovery of AR27 dye and for the reduction of operating costs in the treatment process.

**Fig. 9:**
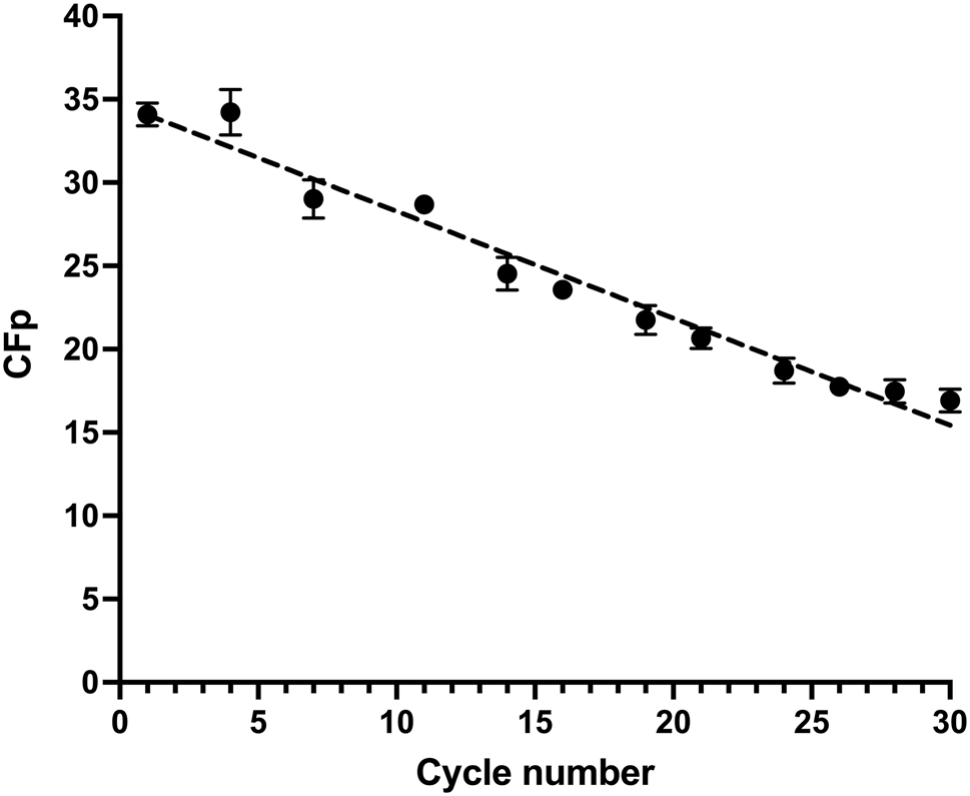
Concentration factor as a function of biosorption/desorption cycle number

The overall process concentration ratio or concentration factor (*C*_Fp_) was used to assess overall biosorption performance. *C*_Fp_ is the ratio of the maximum desorbed dye concentration (*C*_p_) to the dye concentration in the influent (*C*_o_ = 75 mg/L) [29, 37]. *C*_Fp_ is the fold increase in dye concentration relative to the dye concentration in the influent. *C*_Fp_ increases with the amount of dye desorbed within a short time and with decreasing effluent volume. The higher the concentration factor (*C*_Fp_), the greater the feasibility of recovering the dye at higher eluate concentrations, and the greater the overall biosorption performance. It was found that the concentration factor decreased from 34.22 to 16.92 as the number of biosorption/desorption cycles increased from 1 to 30 (Fig. 9).

As all the dye that was initially bound to the AR27-loaded LEC biomass was completely desorbed in all cycles, the observed decrease in concentration factor may be explained by the fact that the overall capacity of the LEC biosorbent decreased with an increasing number of biosorption/desorption cycles.

The *C*_Fp_ values obtained herein were substantially higher than those reported in previous works. Charumathi and Das [31] stated that *C*_Fp_ decreased from 7.70 to 4.14 over eight synthetic dye biosorption/desorption cycles involving dead *Candida tropicalis* cells immobilized on alginate beads. Jafari and Jamali [26] investigated Cd(II) biosorption/desorption using *Sargassum angustifolium* as a biosorbent and found that *C*_Fp_ decreased from 14.22 to 10.72 over four cycles. Martín-Lara et al. [29] reported that *C*_Fp_ decreased from 21.31 to 15.47 over 14 cycles of Pb(II) biosorption/desorption with H_2_SO_4_-pretreated olive stone.

The above results indicate clearly that dilute NaHCO_3_ (0.025 M) can effectively desorb LEC-biosorbed AR27 dye. Subsequently, the LEC can be composted and used as a soil amendment, when its ability to biosorb AR27 has decreased considerably.

## Conclusions

In the present study, we investigated the biosorption of AR27 from aqueous solutions by LEC and the desorption of AR27 from loaded LEC in an up-flow packed-bed column. Desorption tests using 0.025 M NaHCO_3_ as the eluent established that a volumetric flux of 113 L/m^2^·h was optimal for desorbing all AR27 from the loaded LEC in the shortest time. Packed-bed AR27 biosorption and elution curves were plotted for 30 consecutive biosorption/desorption cycles. The properties of LEC biosorption of AR27 were estimated to evaluate biosorbent performance. LEC retained most of its initial biosorption capacity for at least the first 12 biosorption/desorption cycles, retained relatively high biosorption capacity after 30 cycles, and was effectively regenerated each time. In all 30 cycles, 100% of the dye was recovered from the LEC biomass. A life factor analysis based on the biosorption capacity of LEC revealed that the packed bed would be fully depleted after 51.95 cycles. The great ability of LEC to biosorb and desorb AR27 dye makes it promising for the removal of AR27 from wastewater.

## Data Availability

The raw/processed data to support the findings of this study are included in the article.

## Abbreviations

ANOVA: Analysis of variance
AR27: Acid red 27
*C*_Fp_: Concentration factor
*C*_o_: Initial concentration
*C*_p_: Peak concentration
*C*_tD_: Desorbed AR27 concentration
*ε*_b_: Packed bed porosity
*E*_D_: Desorption efficiency
*F*_D_: Desorbent volumetric flow rate
*H*_M__I__N_: Minimum packed-bed height
*k*_b_: H/cycle (life factor)
*k*_H_: Cm/cycle (life factor)
*k*_q_: Mg/g/cycle (life factor)
LEC: Water hyacinth leaves
*m*_D_: Mass of desorbed AR27
*m*_T_: Mass of biosorbed AR27
*q*_b_: Biosorption capacity
*ρ*_b_: Packed bed density
*t*_br_: Breakthrough time
*t*_TD_: Total desorption time
*t*_ex_: Bed exhaustion time
*t*_T_: Total operating time
UV–Vis: Ultraviolet–Visible (spectrum)
